# *Shigella sonnei* bacteraemia in a cystic fibrosis patient: case report and literature review

**DOI:** 10.1099/acmi.0.000102

**Published:** 2020-02-14

**Authors:** Octavio Carretero-Vicario, Irene Taravillo, Laura Corbella, Mercedes Catalan, Cristina Garfia, M. Teresa Martinez, Fernando Chaves, M. Angeles Orellana

**Affiliations:** ^1^​ Department of Microbiology, Hospital Universitario 12 de Octubre, Madrid, Spain; ^2^​ Department of Microbiology, Instituto de Investigación Hospital 12 de Octubre, Madrid, Spain; ^3^​ Unit of Infectious Diseases, Hospital Universitario 12 de Octubre, Madrid, Spain; ^4^​ Intensive Medicine Service, Hospital Universitario 12 de Octubre, Madrid, Spain; ^5^​ Unit of Cystic Fibrosis, Hospital Universitario 12 de Octubre, Madrid, Spain

**Keywords:** bacteraemia, *Shigella sonnei*, *Clostridioides difficile*, coinfection, cystic fibrosis

## Abstract

**>Introduction:**

Shigellosis has a gastrointestinal presentation of variable severity in which bacteraemia is uncommon. We describe the first reported case of *Shigella sonnei* bacteraemia and intestinal coinfection with *Clostridioides difficile* in a cystic fibrosis patient. The literature on *S. sonnei* bacteraemia in adult and paediatric populations is also reviewed.

**Case presentation:**

A 29-year-old male with cystic fibrosis presented with profuse acute watery diarrhoea, abdominal pain, shivering and fever. The patient showed mixed cardiogenic and septic shock. Despite antibiotic therapy, volume replacement therapy and vasoactive drugs, the patient showed biventricular dysfunction and multiple organ failure requiring implantation of an intra-aortic balloon pump (IABP) with extracorporeal membrane oxygenation (ECMO). *C. difficile* and *S. sonnei* were detected in the stools. *Escherichia coli* was identified in the blood by matrix-assisted laser desorption/ionization time-of-flight (MALDI-TOF) mass spectrometry, although after re-evaluation with biochemical and antiserum agglutination tests, the isolate was confirmed as *S. sonnei*. After adjustment of the antibiotic therapy to vancomycin, meropenem, amikacin and metronidazole and continuing with ECMO and IABP support for 8 days, the patient improved and was finally discharged after 44 days.

**Conclusion:**

*S. sonnei* bacteraemia is an unusual entity that should be kept in mind because of the severity of its presentation and high mortality. In acute gastroenteritis and fever, especially in paediatric patients under 5 years old and adults with criteria for immunosuppression or chronic diseases, blood and stool cultures provide simple information that is nonetheless very important for the management and prognosis of these patients.

## Introduction

The genus *Shigella* belongs to the family *Enterobacteriaceae* and comprises four species*: Shigella dysenteriae*, *Shigella flexneri*, *Shigella boydii* and *Shigella sonnei*. Infections vary from asymptomatic presentations and self-limiting gastroenteritis to dysentery with fever, abdominal cramps and blood and/or mucus in diarrhoea [1]. Unlike other members of family *Enterobacteriaceae*, *Shigella* spp. do not penetrate the lamina propria of the intestinal mucosa, so that bacteraemia is very infrequent, especially in the case of *S. boydii* and *S. sonnei* [2]. This report describes a rare case of bacteraemia caused by *S. sonnei* and intestinal coinfection with *Clostridioides difficile* in an adult cystic fibrosis patient treated at our medical centre, and provides a summary of the existing literature on *S. sonnei* bacteraemia in paediatric and adult populations.

## Case report

A 29-year-old Spanish male diagnosed with cystic fibrosis (mutation ∆F508 of the CFTR gene), complicated by exocrine pancreatic insufficiency, bilateral bronchiectasis and repeated respiratory infections, was admitted to the emergency department. The patient reported 12 h of profuse diarrhoea with up to 10–15 liquid stools without mucus, blood or other pathological products, and had abdominal pain, sporadic nausea and vomiting, fever of 39 °C and shivering.

The physical examination showed a body temperature of 38.6 °C, blood pressure of 63/30 mmHg, heart rate of 150 b.p.m. and data for peripheral hypoperfusion with delayed capillary refill. The patient also showed dry mucous membranes, decreased abdominal sounds and generalized pain with no signs of peritoneal irritation. The laboratory results revealed: white blood cell count 8400 cells µl^−1^ (90 % neutrophils), C-reactive protein 23.13 mg dL^−1^, procalcitonin 302.68 ng ml^−1^, glycaemia 240 mg dL^−1^, serum creatinine 3.41 mg dL^−1^ and glomerular filtration rate 29 ml/min/1.73 m^2^. The venous blood gas analysis was: pH 7.25, HCO3 14 mmol l^−1^, pCO2 31 mmHg and lactate 9.80 mmol l^−1^, indicating metabolic acidosis. Amylase and liver function tests were normal. The diagnosis was mixed septic and cardiogenic shock with an abdominal focus, and secondary acute renal failure.

In the emergency room, intensive volume replacement was started, administering up to 2000 ml of saline solution. Empirical antibiotic therapy with ceftriaxone IV and metronidazole IV was also started. As the patient did not respond, he was transferred to the intensive care unit (ICU).

Upon arrival in the ICU, the APACHE II severity score was 32 and the SAPS II score was 53, and the patient required orotracheal intubation with mechanical ventilation for 4 days. In addition, he received continuous serum perfusion, norepinephrine (0.25 µg/kg min^−1^) and continued with antibiotic therapy. Transthoracic echocardiography showed severe biventricular dysfunction and dobutamine 10 µg/kg min^−1^ and vasopressin 0.7 µg/kg min^−1^ were added. Despite this, the patient remained in shock with multiorgan failure, so that femoro-femoral veno-arterial extracorporeal membrane oxygenation (VA-ECMO) together with an intra-aortic balloon pump (IABP) were put in place for 8 days. Antimicrobial therapy was changed to meropenem plus metronidazole plus amikacin.

Samples for stool culture, *C. difficile* toxin detection and blood cultures were taken and sent to the Microbiology Laboratory prior to antibiotherapy.

Toxigenic *C. difficile* was first detected in stool samples with the C. diff Quik Chek complete enzyme immunoassay (Alere, Waltham, MA, USA), and then confirmed by PCR GeneXpert C. difficile (Cepheid, Sunnyvale, CA, USA). The BACT/ALERT Virtuo system (bioMérieux, Marcy-l’Etoile, France) gave a positive signal in two blood cultures after 24 h of incubation, and Gram staining showed Gram-negative bacilli. After sowing on blood agar plates and 24 h of incubation at 37 °C in both aerobic and anaerobic atmospheres, some greyish bacterial colonies were isolated, which were identified as *Escherichia coli* by matrix-assisted laser desorption/ionization time-of-flight (MALDI-TOF) mass spectrometry (Bruker Daltonics, Billerica, MA, USA), with a score of 2.326.

Simultaneously, the stool culture grew non-lactose-fermenting colonies in selective *Salmonella*/*Shigella* agar (SS) after 24 h of incubation at 37 °C (bioMérieux, Marcy-l’Etoile, France). The MicroScan WalkAway system (Beckman Coulter, Brea, CA, USA) identified them as *S. sonnei*, which was confirmed using Shigella Antisera Poly (BD Difco, Franklin Lakes, NJ, USA) for identification of somatic (O) antigens. The susceptibility results, according to the European Committee on Antimicrobial Susceptibility Testing (EUCAST) breakpoint v. 8.0, were: ceftriaxone <1 µg ml^−1^ (S), meropenem <1 µg ml^−1^ (S), ampicillin >16 µg ml^−1^ (R), ciprofloxacin >2 µg ml^−1^ (R) and trimethoprim/sulfamethoxazole >2/38 µg ml^−1^ (R).

Finally, the MicroScan Walkaway system identified *E. coli* in the blood culture as *S. sonnei,* with the same susceptibility pattern as in stool. Identification was confirmed by agglutination with Shigella Antisera Poly.

Immunological tests were performed, which showed HIV-negative and normal lymphocyte populations. The main findings were IgA 651 mg dl^−1^ (70–400), IgG4 262 mg dL^−1^ (3–210) and complement activity 104.17 U ml^−1^ (41.68–95.06).

The patient was treated with vancomycin 500 mg 6 h^−1^ administered by nasogastric tube, meropenem 2 g 8 h^−1^ i.v. in extended infusion, a single dose of amikacin 1 g i.v. and metronidazole 500 mg 6 h^−1^, also administered by nasogastric tube, following Centers for Disease Control and Prevention (CDC) guidelines for the treatment of *Shigella* infections [3]. On the ninth day of admission, the haemodynamic, renal and infectious status of the patient improved and ECMO, IABP and vasoactive support were removed. After 14 days of admission in the ICU, the patient was transferred to the internal medicine unit. Although he was clinically stable, he had ischemic necrotic lesions on the distal phalanges of the hands and feet associated with hypoperfusion during septic and cardiogenic shock. Meropenem and metronidazole were removed and vancomycin was continued. The patient evolved favourably and was finally discharged 44 days after admission; nevertheless, amputation of various phalanges in the feet and hands was necessary because of the risk of necrotizing soft tissue infection.

## Discussion

*Shigella* bacteraemia is a very rare condition. Its prevalence is estimated at 0.4–7.3 % of *Shigella* infections in the adult population [4, 5] and the mortality rate is 21 % [2]. Risk factors predisposing to an invasive presentation are immunodeficiency, diabetes, leukaemia, sickle cell disease, malignancies, HIV, cirrhosis, alteration of intestinal integrity and transplantation [2, 6–12]. *Shigella* bacteraemia is more frequent in the paediatric population, with a prevalence of 5–12 % of all infections [2], accompanied by higher mortality than in adults, reaching 46 % [13], especially in malnourished children in countries with very poor socio-economic conditions [7, 14].

The species most frequently found in bacteraemia are *S. flexneri* and *S. dysenteriae*, with the first being the most virulent [2]. Although all *Shigella* species produce the plasmid-encoded enterotoxin, ShET2 [15], and *S. flexneri* produces the chromosomal enterotoxin, ShET1 [16], only the Shiga toxin has been shown to play an important role in the onset of the pathology. This toxin is generated by *S. dysenteriae*, and in specific cases by *S. sonnei* and *S. flexneri* [17, 18]. Nevertheless, according to the literature, *Shigella* bacteraemia is less likely to be due to the toxin or other virulence factors in specific strains than to patient comorbidities that predispose to systemic invasion [2, 19, 20].

The scientific literature was reviewed and 43 documented cases of *S. sonnei* bacteraemia were found containing demographic, clinical and microbiological data (Table 1). Of those 43 cases, 20 were in children (46.5 %) and 23 were in adults (53.5 %). Nineteen of the 20 paediatric patients were under 5 years old (95 %) and 12 (60 %) presented some risk factor, with malnutrition being the most common (5 patients), followed by maternal infection caused by *S. sonnei* prior to birth (4 patients), sickle cell anaemia (2 patients) and acute lymphoid leukaemia (1 patient). Mortality was 20 %, which was considerably lower than the 46 % mortality previously attributed to bacteraemia caused by the genus *Shigella* in the paediatric population [13]. This could be explained by the lower virulence of S. *sonnei* relative to *S. flexneri* and *S. dysenteriae*, the main causative organisms of bacteraemia [2]. Of the 23 adult cases, 18 (78.3 %) had risk factors such as diabetes (5 patients), malignancies (4 patients), AIDS (3 patients) or solid organ transplantation (1 patient). Some had significant immunosuppression and severity scores, which helped raise the mortality rate to 30.4 %, *a priori* higher than the estimated 21 % for bacteraemia caused by the genus *Shigella* in adults [2].

**Table 1.** Reported cases of *Shigella sonnei* bacteraemia

Authors/year/reference	Patient no./age/ sex	Risk factor	Type of diarrhoea	Outcome
Tatham *et al*. 1951 [22]	1/2/M	None	B*, M*	Recovered
Winter *et al*. 1962 [23]	2/25/M	None	w*	Recovered
Johnston *et al*. 1964 [24]	3/9 m/M	None	ns*	Recovered
Whitfield *et al*. 1967 [25]	4/3d/M	Mother asymptomatic carrier of *S. sonnei*	No diarrhoea*	Recovered
Kraybill *et al*. 1968 [26]	5/2d/M	16 years old, mother with *S. sonnei* diarrhoea before birth	B*	Recovered
Rubin *et al*. 1968 [27]	6/2/F	Sickle-cell anaemia	No diarrhoea	Recovered
Barrett-Connor *et al*. 1969 [28]	7/1/M	None	w*	Recovered
	8/4/F	None	w*	Recovered
Evans *et al*. 1972 [29]	9/2/M	Sickle-cell anaemia	w*	Recovered
Fernhof *et al*. 1973 [30]	10/3/M	None	w*	Recovered
Neter *et al*. 1974 [31]	11/29/M	Renal transplantation	ns*	Recovered
Moore 1974 [32]	12/1d/F	Mother with *S. sonnei* diarrhoea before birth	ns*	Died
Spiers 1974 [33]	13/3/F	Acute monocytic leukaemia	ns*	Died
Scragg *et al*. 1978 [13]	14/6 m/NS	Marasmus	w*	Recovered
	15/1/NS	Marasmus	w*	Recovered
Aldrich *et al*. 1979 [34]	16/2d/M	Malnourished	ns*	Died
Barton et al. 1980 [35]	17/9 m/F	None	w*	Recovered
O´Connor et al. 1981 [36]	18/73/F	None	B*, M*	Died
Roncoroni *et al*. 1984 [37]	19/NS/NS	Chronic kidney disease	ns*	Recovered
	20/NS/NS	None	ns*	Died
Kligler *et al*. 1984 [38]	21/35/M	None	B*	Recovered
	22/17/M	None	w*	Recovered
Schmilovitz *et al*. 1985 [39]	23/58/F	Diabetes mellitus and corticoid therapy	ns*	Died
Alkan *et al*. 1985 [40]	24/53/M	Liver adenocarcinoma and peritoneum	No diarrhoea	Died
Ruderman et al. 1986 [41]	25/2d/M	17 years old, mother with *S. sonnei* diarrhoea before birth	No diarrhoea	Recovered
Whimbey *et al*. 1986 [42]	26/NS/NS	AIDS	w*	Recovered
Morduchowicz *et al*. 1987 [7]	27/88/F	Diabetes mellitus	B*, M*	Recovered
	28/19/F	None	w*	Recovered
Dronda *et al*. 1988 [43]	29/65/F	Diabetes mellitus	w*	Recovered
Christensen *et al*. 1990 [44]	30/34/F	Congenital antithrombin III deficiency and splenectomy	ns*	Recovered
Seymour *et al*. 1994 [19]	33/32/M	Chronic HBV and AIDS	w*	Died (cryptococcal meningitis)
Kenet *et al*. 1994 [10]	34/43/F	Metastatic breast carcinoma and neutropenia	ns*	Died
Ben Salas *et al*. 1995 [45]	35/3 m/M	Premature birth and malnourished	M*	Died
Hawkins *et al*. 2007 [46]	36/65/M	Multiple myeloma and haematopoietic stem cell transplantation	ns*	Recovered
	37/69/M	Diabetes mellitus	w*	Recovered
Liu *et al*. 2009 [47]	38/62/M	Diabetes mellitus and lung cancer	No diarrhoea	Died
Markham *et al*. 2012 [48]	39/31/F	AIDS and pregnant	No diarrhoea	Recovered
Huynh *et al*. 2015 [49]	40/34/M	Risky sexual relations with other men	w*	Recovered
Shogbesan *et al*. 2017 [50]	41/27/M	Risky sexual relations with other men and gastric bypass for morbid obesity 9 years previously	M*	Recovered
Nayyar *et al*. 2017 [6]	42/6 m/M	Malnourished	w*	Recovered

*m, months; d, days; M, male; F, female; ns, not stated; B, bloody; M, mucoid; w, watery.

Almost all the cases of *S. sonnei* bacteraemia described presented with previous diarrhoea, with the exception of six patients: three children and three adults. The most common type of diarrhoea in both populations was watery diarrhoea, rather than diarrhoea with the presence of blood and/or mucus, which is typical of intestinal shigellosis [1].

To the best of our knowledge, this is the first reported case of *Shigella* bacteraemia in a cystic fibrosis patient coinfected with *C. difficile*. A possible cause of this bacteraemia may have been malnutrition, since our patient had exocrine pancreatic insufficiency as a complication of cystic fibrosis. Some authors [2, 13, 20] have reported that malnutrition may facilitate an invasive presentation because it produces decreased secretion of immunoglobulins, complement and other proteins involved in opsonization and lysis of micro-organisms, as well as increasing transferrin saturation [2, 20]. The normal immunological values obtained in our patient, however, would seem to rule out the hypothesis of malnutrition as facilitating this presentation. On the other hand, coinfection with *C. difficile* would probably contribute to intestinal barrier damage and facilitate the translocation of *S. sonnei* to the blood. The association of the two toxins (*C.difficile* and *S. sonnei*) would probably have exacerbated the seriousness of the case, leading to septic and cardiogenic shock and acute renal failure in the patient.

With regard to the microbiological diagnosis, a limitation of MALDI-TOF mass spectrometry is its inability to identify the genus *Shigella* and differentiate it from *E. coli,* because the genera *Escherichia* and *Shigella* are practically identical at the ribosomal protein level [21]. As a result, it is necessary to use traditional diagnostic techniques, such as biochemical and serological tests or sequencing, for identification.

In conclusion, it is important to emphasize that although *S. sonnei* bacteraemia is a rare entity, it should be borne in mind because of the severity of its presentation and high mortality. Therefore, in acute gastroenteritis and fever, especially in paediatric patients under 5 years old and adults with criteria for immunosuppression or chronic diseases, blood and stool cultures provide simple information that is very important for the management and prognosis of these patients.
